# Identification and analysis of chemokine-related and NETosis-related genes in acute pancreatitis to develop a predictive model

**DOI:** 10.3389/fgene.2024.1389936

**Published:** 2024-05-09

**Authors:** Shuangyang Mo, Wenhong Wu, Kai Luo, Cheng Huang, Yingwei Wang, Heping Qin, Huaiyang Cai

**Affiliations:** ^1^ Gastroenterology Department, Liuzhou People’s Hospital Affiliated to Guangxi Medical University, Liuzhou, China; ^2^ Department of Critical Care Medicine, Liuzhou People’s Hospital Affiliated to Guangxi Medical University, Liuzhou, China; ^3^ Oncology Department, Liuzhou People’s Hospital Affiliated to Guangxi Medical University, Liuzhou, China

**Keywords:** acute pancreatitis, NEtosis, chemokine, machine learning, nomogram, bioinformatics

## Abstract

**Background:** Chemokines and NETosis are significant contributors to the inflammatory response, yet there still needs to be a more comprehensive understanding regarding the specific molecular characteristics and interactions of NETosis and chemokines in the context of acute pancreatitis (AP) and severe AP (SAP).

**Methods:** To address this gap, the mRNA expression profile dataset GSE194331 was utilized for analysis, comprising 87 AP samples (77 non-SAP and 10 SAP) and 32 healthy control samples. Enrichment analyses were conducted for differentially expressed chemokine-related genes (DECRGs) and NETosis-related genes (DENRGs). Three machine-learning algorithms were used for the identification of signature genes, which were subsequently utilized in the development and validation of nomogram diagnostic models for the prediction of AP and SAP. Furthermore, single-gene Gene Set Enrichment Analysis (GSEA) and Gene Set Variation Analysis (GSVA) were performed. Lastly, an interaction network for the identified signature genes was constructed.

**Results:** We identified 12 DECRGs and 7 DENRGs, and enrichment analyses indicated they were primarily enriched in cytokine-cytokine receptor interaction, chemokine signaling pathway, TNF signaling pathway, and T cell receptor signaling pathway. Moreover, these machine learning algorithms finally recognized three signature genes (S100A8, AIF1, and IL18). Utilizing the identified signature genes, we developed nomogram models with high predictive accuracy for AP and differentiation of SAP from non-SAP, as demonstrated by area under the curve (AUC) values of 0.968 (95% CI 0.937–0.990) and 0.862 (95% CI 0.742–0.955), respectively, in receiver operating characteristic (ROC) curve analysis. Subsequent single-gene GESA and GSVA indicated a significant positive correlation between these signature genes and the proteasome complex. At the same time, a negative association was observed with the Th1 and Th2 cell differentiation signaling pathways.

**Conclusion:** We have identified three genes (S100A8, AIF1, and IL18) related to chemokines and NETosis, and have developed accurate diagnostic models that might provide a novel method for diagnosing AP and differentiating between severe and non-severe cases.

## Introduction

Acute pancreatitis (AP) is one of the most common abdominal acute conditions (Liang et al., 2021; Sheng et al., 2021). The 2012 revised Atlanta Classification divides acute pancreatitis into three categories: mild acute pancreatitis (MAP), moderately severe acute pancreatitis (MSAP), and severe acute pancreatitis (SAP) (Zhao et al., 2023). The outcome of acute pancreatitis is determined by the seriousness of the condition (Lin et al., 2019). SAP accounts for approximately 20% of AP cases (Zhao et al., 2021). MAP presents with local inflammation, whereas SAP is characterized by systemic inflammatory response syndrome, which is associated with a 30% mortality rate (Peng et al., 2023). Symptoms of SAP include persistent organ failure (>48 h) of the respiratory, cardiovascular, and renal systems due to cytokine cascade-induced systemic inflammation (Akiode et al., 2022). Inflammation and cell death are two AP hallmarks (Zhan et al., 2019). Moreover, inflammatory factors and their cascade reaction and uncontrolled inflammation will lead to SAP. In patients with SAP, inhibiting the release of inflammatory factors is the top priority of treatment (Yang et al., 2022).

AP is characterized by edema, necrosis, and neutrophilic infiltrates histologically (Hardwick et al., 2022). The neutrophils are the prominent actors in the acute inflammation of AP, playing critical components of SAP and releasing cytokines that activate the inflammatory responses of several other immune cells (Murthy et al., 2019; Andersson et al., 2020). Neutrophils, as the primary responders and essential components of the innate immune system, generate neutrophil extracellular traps (NETs) during acute inflammation. These heterogeneous and plentiful leukocytes play a crucial role in acute inflammatory responses, such as infection and injury (Han et al., 2022). In a recent study, NETs were identified as an effector function of neutrophils, characterized by extracellular networks of chromatin coated with histones, myeloperoxidase, and neutrophil elastase (Colón et al., 2019; Zhang et al., 2020). Neutrophils carry out their physiological roles through mechanisms such as phagocytosis, degranulation, and the release of NETs (Muqaku et al., 2020). The process of NET formation has been identified as a novel form of neutrophil cell death known as NETosis (Cho and Cho, 2019; Chen et al., 2022).

In recent years, NETosis research has been booming (Wang H. et al., 2023). Evidence shows that dysregulated NETosis leads to hypercoagulability and thrombosis (Dechamps et al., 2021). Notably, neutrophil influx and cancer cells can release chemokines that attract neutrophils and induce NETs formation (Wang H. et al., 2023). The pathophysiological role of NETosis in neutrophil infection immunity is not entirely understood, but it is an essential mechanism for neutrophil anti-infection immunity (Ou et al., 2021). Despite the crucial role that NETosis plays in antimicrobial processes, excessive NETosis can lead to severe tissue damage and exacerbation of inflammation (Hafkamp et al., 2022). It is noteworthy that NETosis causes thrombosis, which exacerbates COVID-19, cancer, acute myocardial infarction, and ischemic stroke (Wang H. et al., 2023). When chemokines activate neutrophils, they migrate into inflammation sites, produce antimicrobial agents, undergo NETosis, and kill bacteria (Balachandran et al., 2022). An essential function of chemokines is to increase neutrophils and other immune cell recruitment from the peripheral blood to injured tissue (Kong et al., 2022), and previous studies also reported that chemokines play crucial roles in AP (Ramnath et al., 2008). Together, it indicated that the NETosis and chemokine might play a central regulatory intermediary and executor in AP. However, our understanding of the specific molecule character and interaction of NETosis and chemokines within AP and SAP is limited. Hence, a novel predictive diagnostic model for AP, especially SAP, is urgently needed based on molecular mechanisms of NETosis and chemokine-related genes.

In contemporary life science research, bioinformatics plays a crucial role facilitated by advancements in high-throughput sequencing and microarray technologies. This analytical tool is utilized to analyze differentially expressed mRNA and predict potential therapeutic targets in specific diseases. Bioinformatic analysis serves as an effective method for identifying biomarkers and elucidating the etiopathogenesis of diseases, thereby offering a valuable foundation for subsequent investigations (Peng et al., 2022). In this study, the Gene Expression Omnibus (GEO) (Clough and Barrett, 2016) dataset was analyzed using bioinformatic methods and machine learning techniques to identify signature NETosis-related and chemokine-related genes for AP, as well as SAP. Additionally, a predictive diagnostic model was developed and assessed.

## Material and method

### Data source of microarray

**FIGURE 1:**
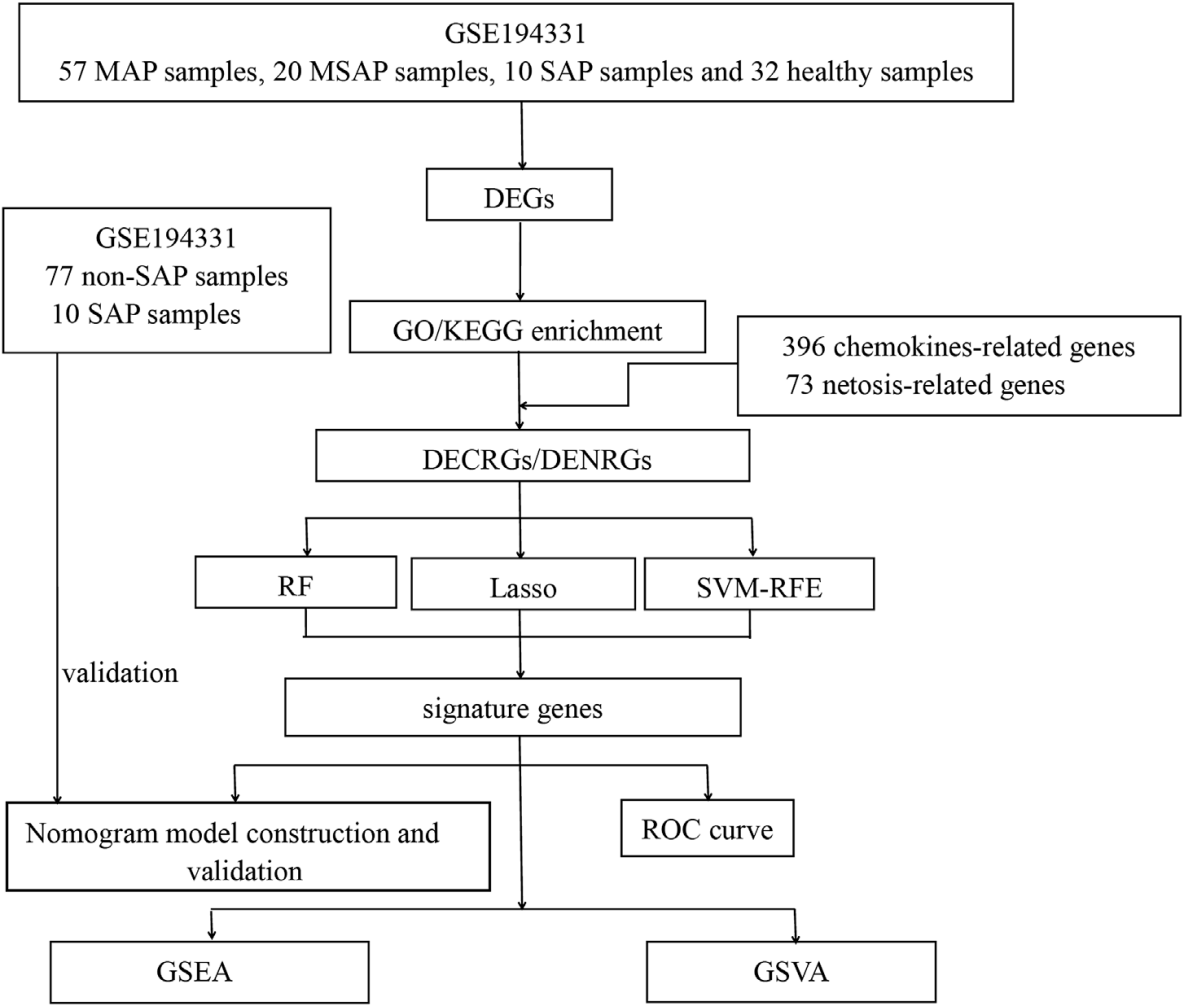
Flowchart of this study. (DEGs, differentially expressed genes; GO, Gene Ontology; KEGG, Kyoto Encyclopedia of Genes and Genomes; DECRGs, differentially expressed chemokine-related genes; DENRGs, differentially expressed NETosis-related genes; RF, random forest; SVM-RFE, Support Vector Machines- Recursive Feature Elimination; LASSO, Least Absolute Shrinkage and Selection Operator; GSEA, Gene Set Enrichment Analysis; GSVA, Gene Set Variation Analysis).

The methodology for data analysis in this study is illustrated in Figure 1. Inclusion criteria were established to ensure that test specimens were sourced from human subjects, focusing on independent expression profiles with the highest sample size. The dataset GSE194331 was selected for inclusion in this research and was obtained from the GEO database. Within this dataset were 87 samples from patients with acute pancreatitis (including 57 with MAP, 20 with MSAP, and 10 with SAP), and 32 samples from healthy individuals, all of whom underwent mRNA expression profiling. The participants were stratified into a training cohort consisting of AP and healthy control samples and an internal validation cohort comprising non-SAP and SAP samples based on the larger sample size of the AP and healthy control groups. Further information can be found in Table 1.

**TABLE 1:** Details of the GEO data.

Dataset	Platform	Number of samples (AP/Healthy control)
GSE194331	GPL16791	119 (87/32)
	Illumina HiSeq 2,500 (*Homo sapiens*)	

GEO, gene expression omnibus.

### Identifying differentially expressed chemokine-related and NETosis-related genes

The differentially expressed genes (DEGs) between samples from individuals with AP and healthy controls were identified using normalized and preprocessed data based on the R limma package. A screening threshold of |log2 Fold Change| >1.0 and *p* < 0.05 was applied. A total of 396 genes involved in the chemokine-related pathway were obtained from the GeneCards database (https://www.genecards.org/), along with 73 genes related to the NETosis pathway. The intersection of DEGs and these chemokine-related genes was defined as the differentially expressed chemokine-related genes (DECRGs). Similarly, the differentially expressed NETosis-related genes (DENRGs) were defined.

## Enrichment analyses of Gene ontology

Kyoto Encyclopedia of Genes and Genomes (KEGG) pathway and Gene ontology (GO) enrichment analyses, encompassing biological process (BP), cellular component (CC), and molecular function (MF), were conducted using the R clusterProfiler package (Dechamps et al., 2021). The false discovery rate (FDR) was determined through Benjamini–Hochberg (BH) adjustment, with a cutoff criterion of q-value <0.05. The significant results of these enrichment analyses were visualized using the R circlize package.

## Machine learning of least absolute shrinkage and selection operator (LASSO), support vector machines- recursive feature elimination (SVM-RFE), and random forest (RF)

Subsequently, machine learning algorithms, precisely the LASSO regression, and SVM-RFE algorithm were employed to identify the feature genes from DECRGs and DENRGs, respectively. The optimal parameter λ for Lasso regression was determined through 10-fold cross-validation using the R glmnet package with the specifications “family = binomial, measure = deviance,” and default settings for all other parameters. Additionally, the SVM-RFE method, known for its effectiveness in feature selection, was utilized to differentiate feature genes from DECRGs and DENRGs with the R e1071 package. The feature DECRGs and DENRGs, derived using LASSO regression and the SVM-RFE algorithm, were incorporated for subsequent analysis.

In the RF algorithm, equipped with a feature selection function, the values of “Mean Decrease Gini” and “Mean Decrease Accuracy” indicate a feature’s importance. The classification process ranks input features DECRGs and DENRGs based on their respective “Mean Decrease Gini” and “Mean Decrease Accuracy” scores, ultimately identifying the top and consistent features as signature genes in the final RF model.

### Establishment of interaction network for signature genes

Subsequently, we constructed an interaction network for signature genes by utilizing GeneMANIA, an online tool for identifying internal correlations within gene sets. The results of this network analysis can be found in Supplementary Material S1.

### Construction of nomogram model and assessment of diagnostic efficacy

Utilizing the R rms package, diagnostic models with nomograms were developed using signature genes. Calibration curves were generated to evaluate the calibration of the nomogram models through mean absolute error and 1,000 bootstrap samples with the R CalibrationCurves package. Decision curve analysis (DCA) was conducted to assess the net benefits of the nomogram models at various high-risk thresholds. Subsequently, the predictive efficacy of the nomogram model was assessed using the clinical impact curve (CIC). Lastly, the receiver operating characteristic (ROC) curve with the area under the curve (AUC) was analyzed to determine the model’s performance. Similarly, a nomogram model based on signature genes was also modeled and validated in the internal validation set.

### Gene set variation analysis and gene set enrichment analysis

Subsequently, our study aims to investigate the potential functions of signature genes in AP. To achieve this, a single-gene Gene Set Enrichment Analysis (GSEA) was conducted for each signature gene using the R clusterProfiler package. Initially, the samples were divided into low-expression and high-expression groups based on the individual expression levels of each signature gene. Subsequently, GSEA was utilized to identify significantly distinct pathways from the KEGG database within these two groups.

The Gene Set Variation Analysis (GSVA) analysis was conducted to illustrate the varying enrichment of KEGG pathways and GO terms among the groups in a comparable manner. The R GSVA package was employed in this investigation using gene sets sourced from c2.cp.kegg.symbols.gmt and c5.go.symbols.gmt from the official repository. A significance threshold of *p* < 0.05 was established for identifying statistically significant terms.

## Results

### Recognition of DEGs

**FIGURE 2:**
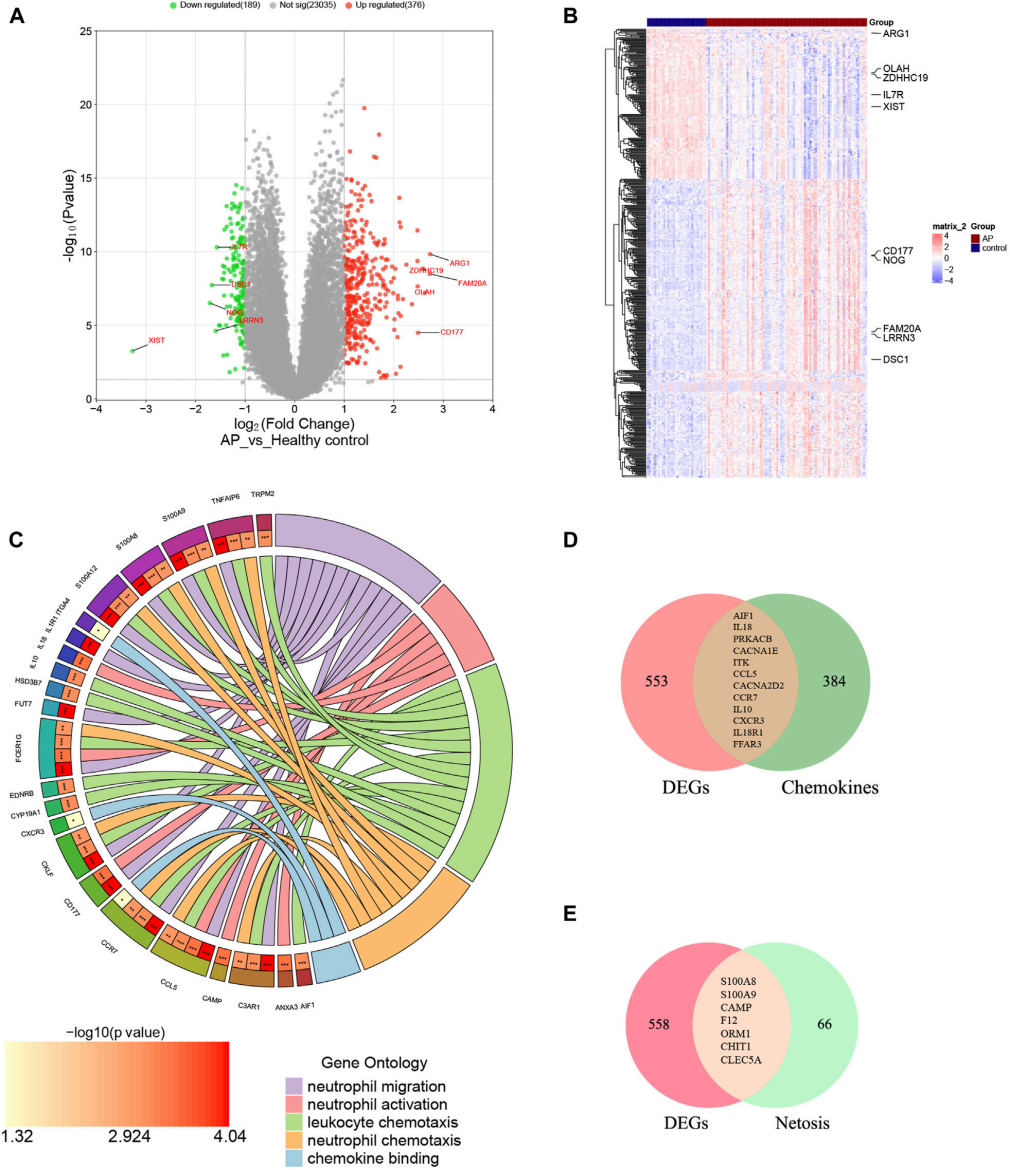
Identification of DECRGs and DENRGs between AP and healthy control groups. **(A)** The volcano plot of DEGs; **(B)** The heatmap of DEGs; **(C)** The chordal graph shows GO enrichment significance items of DEGs between AP and healthy control groups (each string represents the association of DEGs with special GO terms); **(D)** The Venn diagram for recognizing DECRGs (The red region means DEGs, green region means chemokine-related genes and yellow region means DECRGs); **(E)** The Venn diagram for recognizing DENRGs (The red region means DEGs, green region means Netosis-related genes and yellow region means DENRGs).

The mRNA expression profile dataset GSE194331 underwent normalization, identifying 565 DEGs between AP and healthy control groups, consisting of 376 upregulated DEGs and 189 downregulated DEGs. Subsequently, a volcano plot and a heatmap were generated and presented in Figure 2A, B, respectively. The top five most significantly upregulated and downregulated genes (based on log2 Fold Change) were respectively annotated in Figure 2A, B.

### Function enrichment analyses of the DEGs

The biological functions of DEGs were analyzed using GO analyses to enhance understanding. The DEGs were categorized into three functional groups within the GO framework: BP (including 41 DEGs), CC (including 26 DEGs), and MF (including 17 DEGs). The major functional processes of DEGs were lymphocyte differentiation, mononuclear cell differentiation, regulation of inflammatory response, activation of immune response, and positive regulation of cytokine production. These outcomes demonstrated that the inflammatory response and cytokine might be crucial in AP. Furthermore, we found that many DEGs were also significantly involved in processes such as neutrophil migration (including 12 DEGs), neutrophil activation (including 6 DEGs), neutrophil chemotaxis (including 15 DEGs), chemokine binding (including 9 DEGs), and leukocyte chemotaxis (including 3 DEGs), indicating the functions of neutrophils and chemokines are significantly changed in AP, as depicted in Figure 2C and Supplementary Material S2. These results suggest that neutrophil activity and chemokines may be crucial in regulating AP.

### Recognition of DECRGs and DENRGs

Supplementary Materials S3, 4 display the 396 genes involved in the chemokine-related pathway and the 73 genes related to NETosis. From the GSE194331 dataset, a total of 12 DECRGs were identified, following the intersection of DEGs and 396 chemokine-related genes (Figure 2D). Similarly, 7 DENRGs were recognized, following the intersection of DEGs and 73 Netosis-related genes (Figure 2E). As shown in Figure 2C, 14 DEGs were enriched in neutrophil-related functions, of which nine were from DECRGs and DENRGs, indicating that DECRGs and DENRGs might play a vital role in regulating neutrophil activity. Then, all the DECRGs and DENRGs are displayed in Table 2, and their log_2_ Fold Change and *p*-values are shown in Supplementary Material S5.

**TABLE 2:** The DECRGs and DENRGs of GSE194331.

Category	Genes
DECRGs (n = 12)	AIF1, IL18, PRKACB, CACNA1E, ITK, CCL5, CACNA2D2, CCR7, IL10, CXCR3, IL18R1, FFAR3
DENRGs (n = 7)	S100A8, S100A9, CAMP, F12, ORM1, CHIT1, CLEC5A

DECRGs, differently expressed chemokine-related genes; DENRGs, differently expressed Netosis-related genes.

**FIGURE 3:**
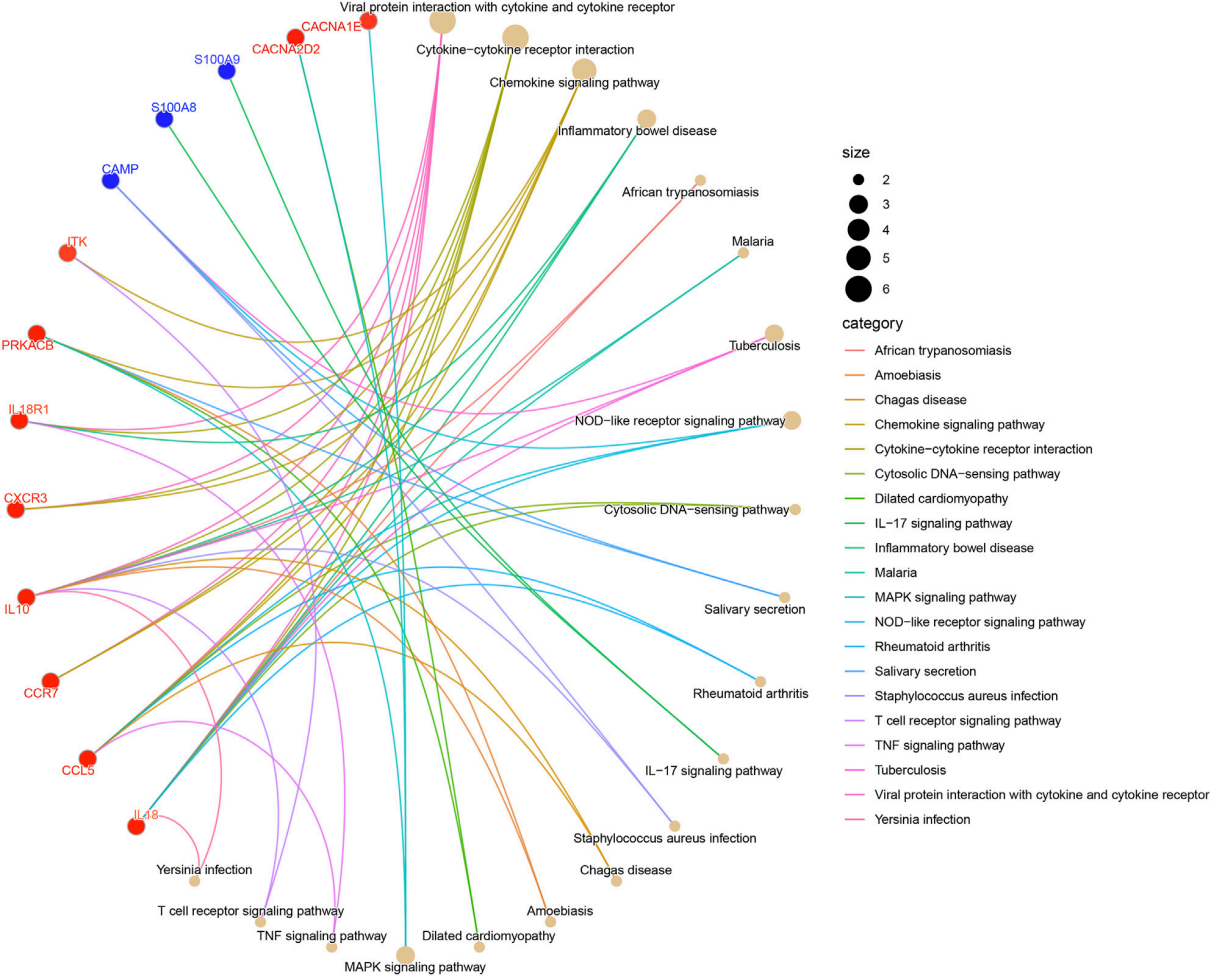
The KEGG pathway enrichment analysis of DECRGs and DENRGs. (Red fonts and dots indicate DECRGs; blue fonts and dots indicate DENRGs).

The outcomes of KEGG pathways enrichment analysis showed that the DECRGs and DENRGs mostly correlated with the cytokine-cytokine receptor interaction, chemokine signaling pathway, NOD-like receptor signaling pathway, IL-17 signaling pathway, MAPK signaling pathway, TNF signaling pathway, and T cell receptor signaling pathway (Figure 3; Supplementary Figure S1).

### The machine learning algorithm of LASSO and SVM-RFE

**FIGURE 4:**
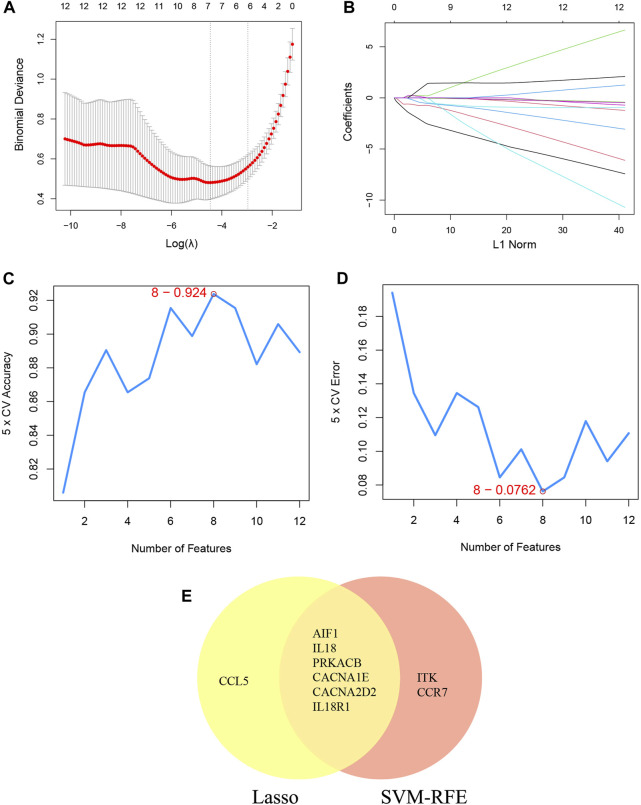
Machine learning for DECRGs. **(A)** The log(λ) value was optimally selected by 10-fold cross-validation and plotted by the partial likelihood deviance; **(B)** Processes of lasso regression for identifying variables and mapping each variable to a curve; **(C)** The accuracy (5× CV) is highest as 0.924 when the number of feature genes is 8; **(D)** The error (5× CV) is lowest as 0.076 when the number of feature genes is 8; **(E)** The Venn diagram for recognizing feature DECRGs.

Additionally, two machine learning algorithms, LASSO and SVM-RFE, were utilized to separately identify feature genes from DECRGs and DENRGs. The LASSO regression, which assumes a linear relationship and incorporates an L1 regularization penalty, was employed. The LASSO regression with the lowest binomial deviance was initially conducted using 10-fold cross-validation. Genes with non-zero regression coefficients were chosen as feature genes for DECRGs. Consequently, a total of 7 DECRGs (CCL5, AIF1, IL18, PRKACB, CACNA1E, CACNA2D2, and IL18R1) were identified in the simplified LASSO regularization model (Figures 4A, B; Table 3).

**TABLE 3:** The feature DECRGs and DENRGs of LASSO and SVM-RFE.

Machine learning	Feature DECRGs	Feature DENRGs
LASSO	AIF1, IL18, PRKACB, CACNA1E, CCL5, CACNA2D2, IL18R1	S100A8, CAMP, F12, ORM1
SVM-RFE	AIF1,PRKACB, ITK, CACNA2D2 IL18, CACNA1E, IL18R1, CCR7	CLEC5A, CHIT1, S100A8, CAMP, F12, ORM1

**FIGURE 5:**
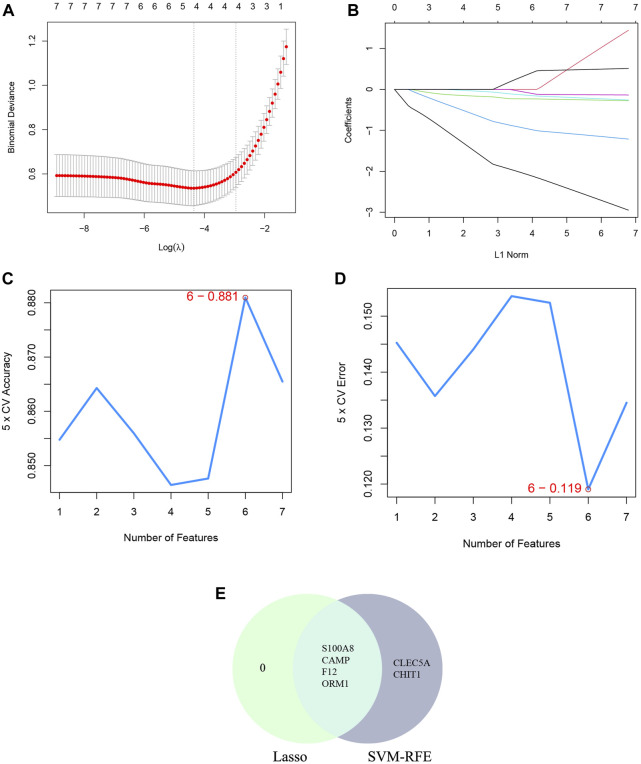
Machine learning for DENRGs. **(A)** the log(λ) value was optimally selected by 10-fold cross-validation and plotted by the partial likelihood deviance; **(B)** Processes of lasso regression for identifying variables and mapping each variable to a curve; **(C)** The accuracy (5× CV) is highest as 0.881 when the number of feature genes is 6; **(D)** The error (5× CV) is lowest as 0.119 when the number of feature genes is 6; **(E)** The Venn diagram for recognizing feature DENRGs.

The SVM-RFE machine learning paradigm, known for its effectiveness in classification, regression, and various machine learning tasks, has frequently demonstrated superior performance compared to other classifiers. Subsequently, the SVM-RFE method was employed to extract optimal feature genes. Upon reaching a count of 8, the model attained a peak accuracy of 92.4% and a minimum error rate of 7.6% concurrently. Consequently, these top 8 DECRGs (ITK, CCR7, AIF1, IL18, PRKACB, CACNA1E, CACNA2D2, and IL18R1) are deemed optimal and utilized for subsequent analyses (Figures 4C, D; Table 3). Subsequently, an intersection was conducted between the two primary gene sets identified by the LASSO and SVM-RFE models, resulting in the identification of 6 feature DECRGs (AIF1, IL18, PRKACB, CACNA1E, CACNA2D2, and IL18R1) (Figure 4E). In addition, following the same screening procedure as DECRGs, we finally retained 4 feature DENRGs (S100A8, CAMP, F12, and ORM1) (Figures 5A–D; Table 3).

### Univariate logistic regression analysis of the feature DECRGs and DENRGs

**FIGURE 6:**
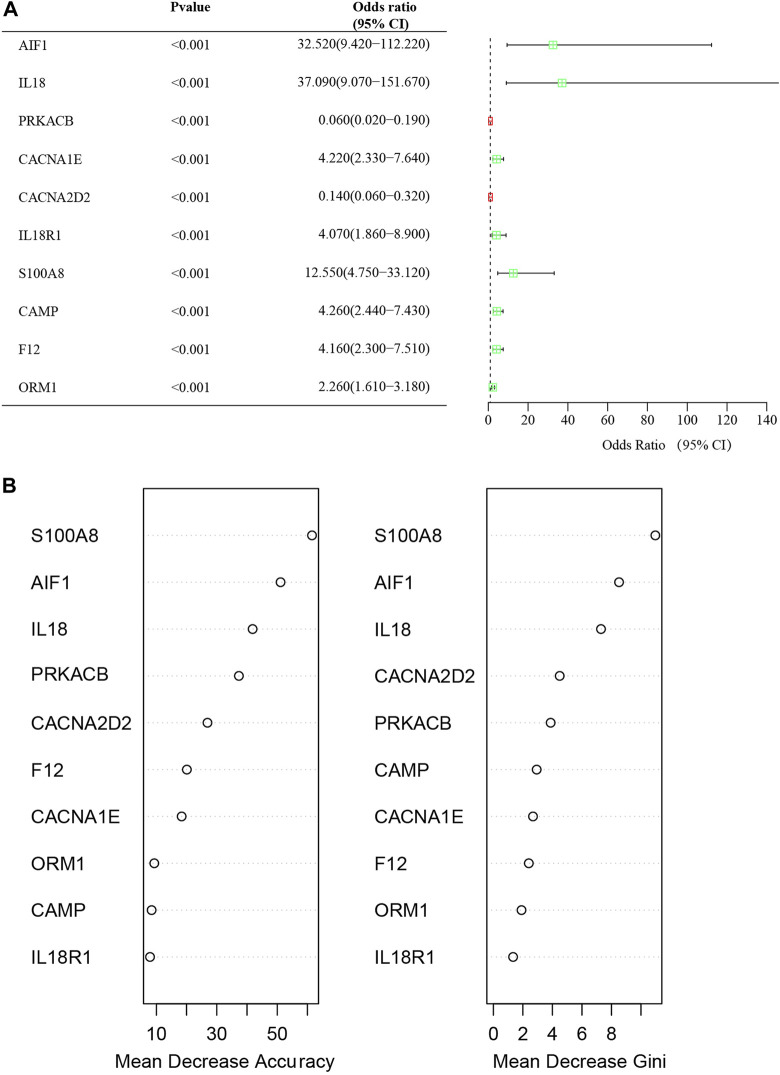
**(A)** Forest map of univariate logistic regression of DECRGs and DENRGs in GSE194331; **(B)** The “Mean Decrease Accuracy” and “Mean Decrease Gini” of each feature DECRGs and DENRGs within RF models for GSE194331.

The study employed univariate logistic regression analyses to assess the relationship between individual DECRGs/DENRGs and the dependent variables of AP *versus* healthy control. Notably, the elevated expression of AIF1, IL18, CACNA1E, IL18R1, S100A8, CAMP, F12, and ORM1 was significantly associated with increased risk of AP, as indicated by odds ratios (OR) greater than 1. Conversely, decreased expression of PRKACB and CACNA2D2 was found to be significantly protective. Forest plots illustrating the risk factors identified through univariate analysis are presented in Figure 6A. These findings suggest that the identified feature DECRGs/DENRGs may play a vital role in the pathogenesis of AP.

### Recognition of signature genes via RF

**FIGURE 7:**
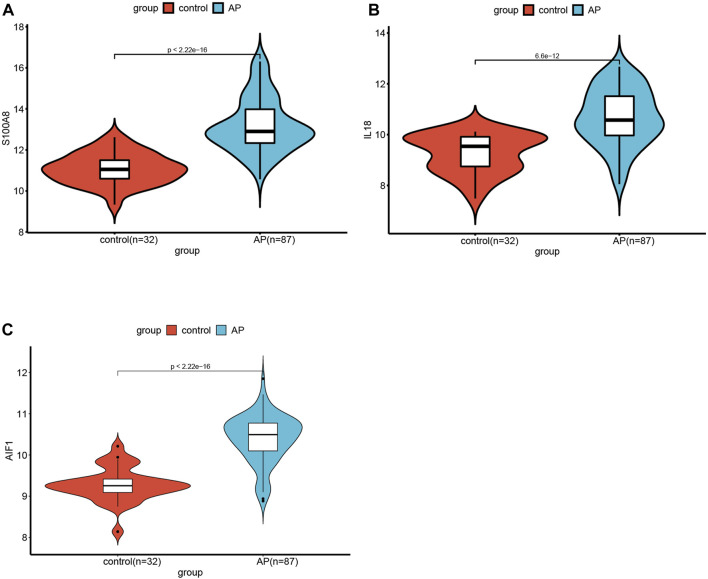
The violin plot of mRNA expression S100A8(A), IL18(B), and AIF(C) in GSE194331 between AP and healthy control groups.

The significance of each feature DECRGs/DENRGs was assessed through the calculation of “Mean Decrease Gini” and “Mean Decrease Accuracy”, resulting in the identification of the top three feature DECRGs/DENRGs consistently selected from the RF model in GSE194331 (Figure 6B). Subsequently, these three feature DECRGs/DENRGs (S100A8, AIF1, and IL18) were collectively designated as the signature genes for AP. Finally, the violin plot of three signature gene expressions between AP and healthy control groups is displayed in Figure 7.

### Construction and assessment of the nomogram model for AP

**FIGURE 8:**
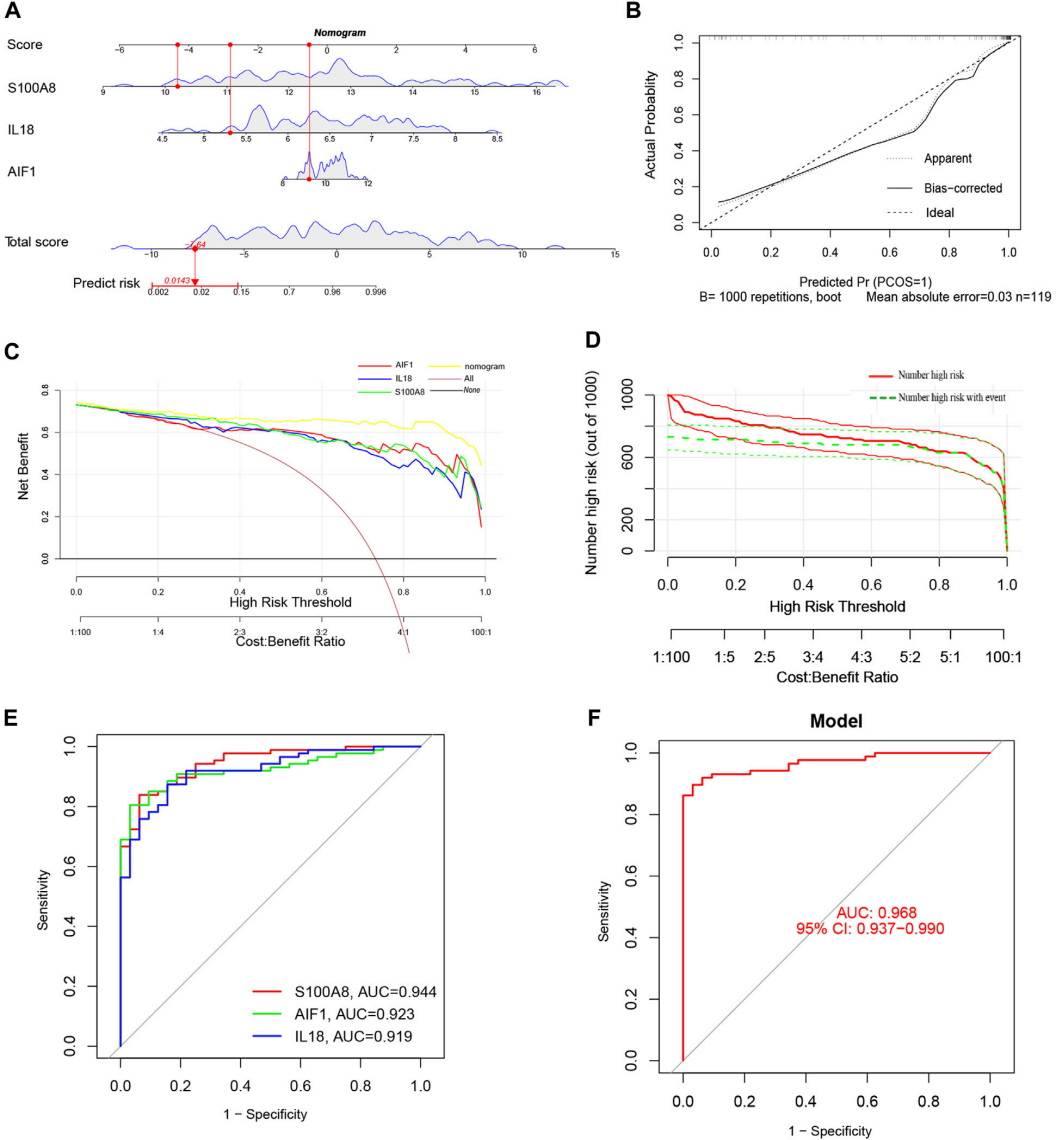
**(A)** The Nomogram model predicting AP based on three signature genes. The nomogram is used by summing all points identified on the scale for each variable. The total points projected on the bottom scales indicate the probabilities of AP; **(B)** The calibration curves for the nomogram with the mean absolute error = 0.03; **(C)** Decision curve analysis (DCA) of the nomogram model and each signature gene (the “All” means diagnosis-all strategy; the “None” means diagnosis-none strategy); **(D)** The clinical impact curve (CIC) of the nomogram model; **(E)** The ROC and AUC of each signature gene between AP and healthy control groups; **(F)** The ROC and AUC of nomogram model between AP and healthy control groups.

Additionally, a nomogram model was developed using logistic regression analysis of three signature genes through the R rms package (Figure 8A). Subsequently, a calibration curve was employed to assess the predictive efficacy of the nomogram model. The calibration curve demonstrated minimal error between the actual and predicted probabilities of AP, with a mean absolute error of 0.03, indicating the high accuracy of this nomogram model in predicting AP (Figure 8B). The findings of the DCA demonstrated that the “Nomogram” curve exhibited higher values compared to the “All” curve, “S100A8” curve, “IL18”curve, “AIF1” curve, and “None” curve within the high-risk threshold ranging from nearly 0 to 1.0. It indicated that patients may experience a net benefit from utilizing the nomogram model (Figure 8C). Additionally, a CIC was constructed based on the DCA curve to evaluate the clinical efficacy of the nomogram model visually. The proximity of the “Number high risk” curve to the “Number high risk with event” curve at a high-risk threshold ranging from 0.2 to 1 suggests that the nomogram model exhibits exceptional predictive capability (Figure 8D). These findings further suggest that the three signature genes may significantly contribute to the pathogenesis of AP.

ROC curves with AUC were utilized to assess the diagnostic efficacy of the nomogram model in distinguishing between individuals with AP and healthy controls based on the expression levels of the identified target genes (S100A8, AIF1, and IL18). The analysis of ROC curves indicated AUC values of 0.944 for S100A8, 0.923 for AIF1, and 0.919 for IL18 (Figure 8E). Furthermore, the AUC for the nomogram model incorporating all three signature genes was calculated to be 0.968 (95% CI, 0.937–0.990) (Figure 8F).

### Construction and assessment of the nomogram model for SAP

**FIGURE 9:**
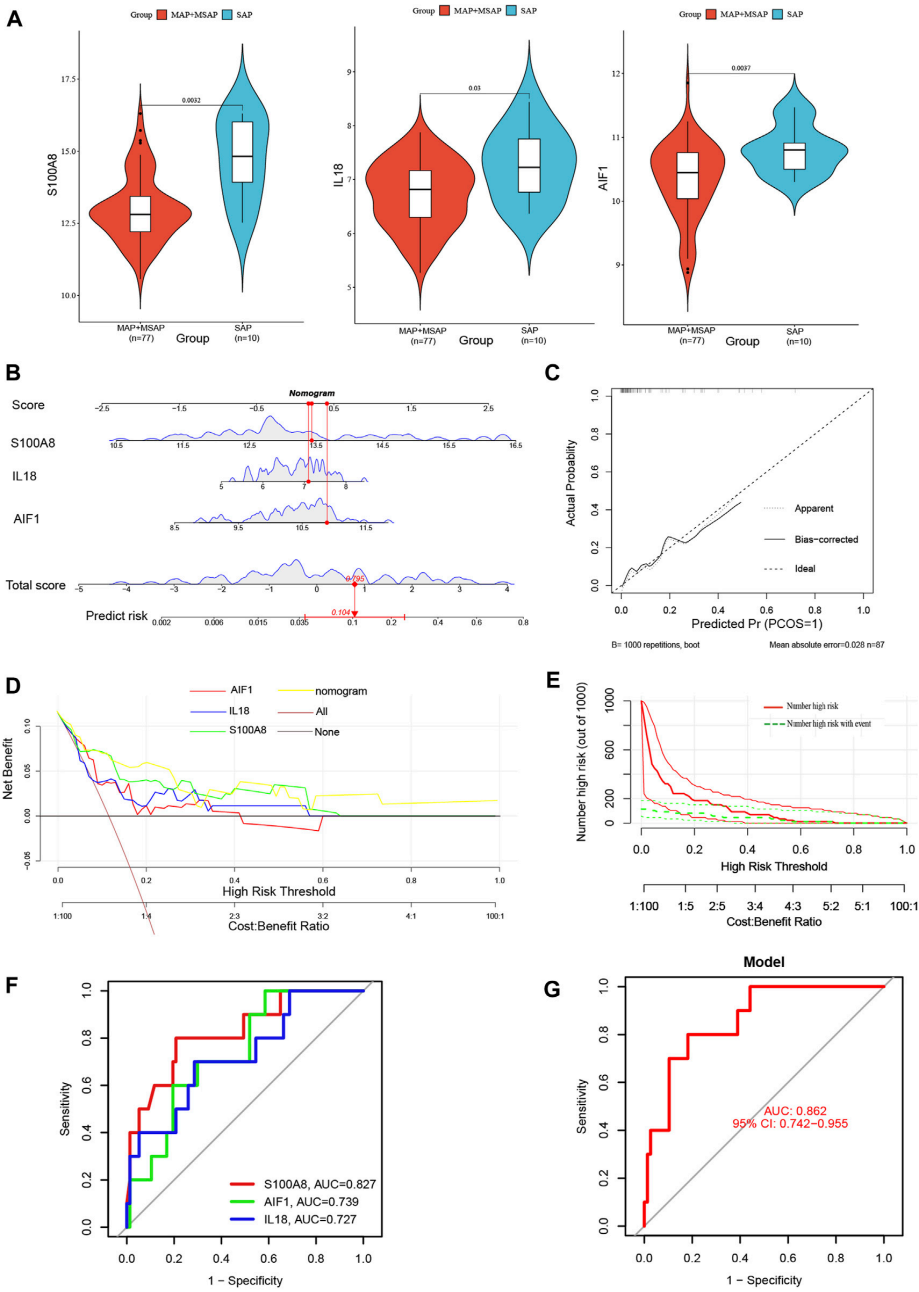
**(A)** The violin plot of mRNA expressionS100A8, IL18, and AIF1 in GSE194331 between SAP and non-SAP groups. **(B)** The Nomogram model predicting SAP based on three signature genes; **(C)** The calibration curves for the nomogram with the mean absolute error = 0.028; **(D)** DCA of the nomogram model and each signature gene (the “All” means diagnosis-all strategy; the “None” means diagnosis-none strategy); **(E)** The CIC of the nomogram model; **(F)** The ROC and AUC of each signature gene between SAP and non-SAP groups; **(G)**The ROC and AUC of nomogram model between SAP and non-SAP groups.

Compared to MAP and MSAP, SAP is distinguished by more pronounced clinical manifestations and a poorer prognosis. Consequently, both SAP and non-SAP (encompassing MAP and MSAP) cohorts were utilized to corroborate the reliability of these three distinctive genes. Notably, the expression levels of these three signature genes were notably elevated in the SAP group (Figure 9A). The nomogram derived from the analysis of these three signature genes is depicted in Figure 9B.

Additionally, this nomogram model demonstrated similarly high accuracy (Figure 9C), net clinical benefit (Figure 9D), and clinical applicability (Figure 9E) for predicting SAP. The analysis of ROC curves indicated AUC values of 0.827 for S100A8, 0.739 for AIF1, and 0.727 for IL18 (Figure 9F). Furthermore, the AUC of the nomogram model for predicting SAP was calculated to be 0.862 (95% CI, 0.742–0.955) (Figure 9G). Summarily, these results suggested that these three signature genes can serve as effective diagnostic biomarkers for predicting AP and distinguishing SAP from non-SAP.

#### Interaction network of signature genes

**FIGURE 10:**
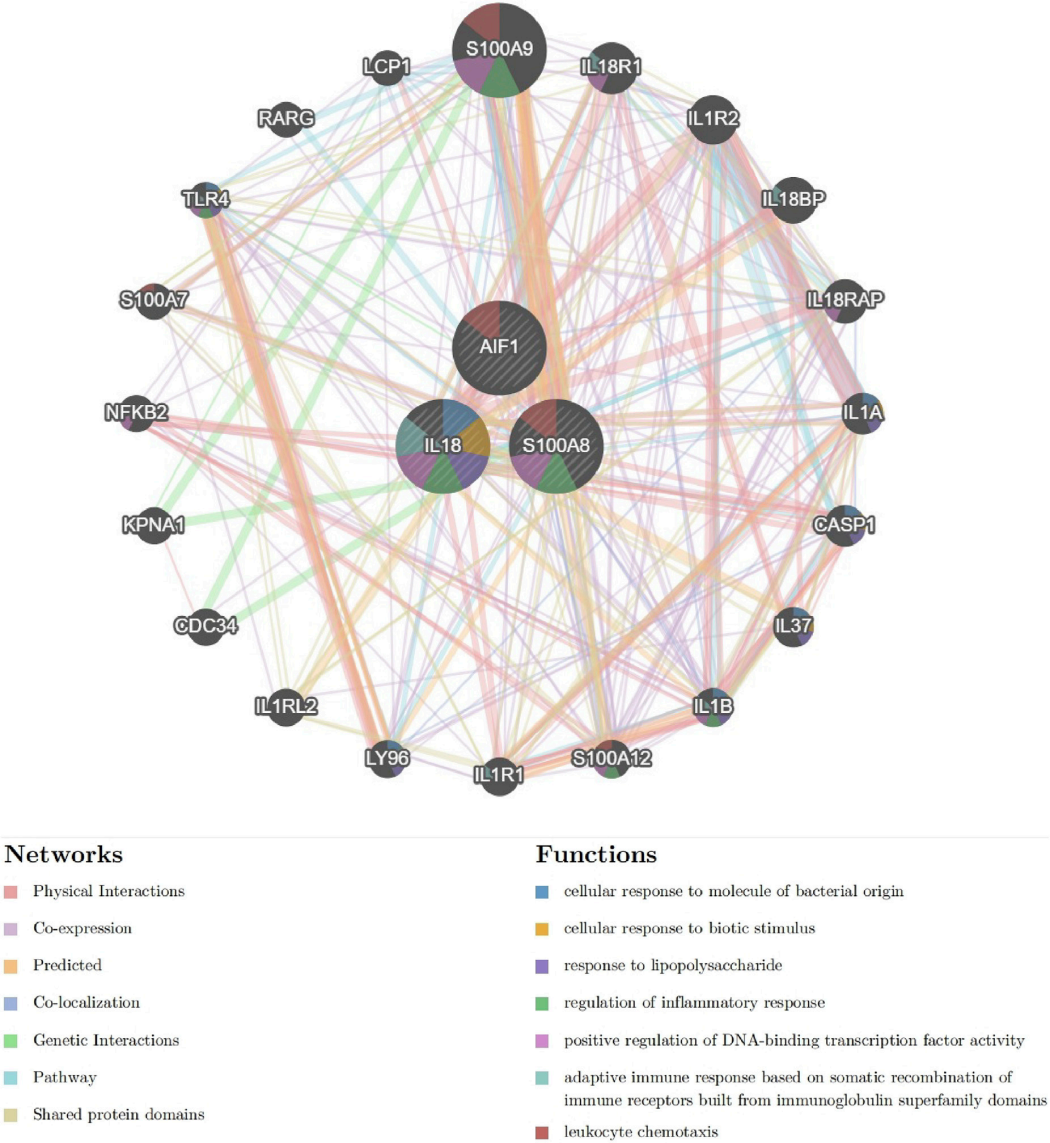
The Interaction network of signature genes via GeneMANIA. (Different line colors indicate the association of different genes and constitute gene networks; Different colors of the gene circle plot represent different gene functions).

The interaction network and associated functions of the identified signature genes were examined. The analysis revealed a multifaceted interaction network consisting of physical interactions (77.64%), co-expression (8.01%), predicted interactions (5.37%), co-localization (3.63%), genetic interactions (2.87%), pathway associations (1.88%), and shared protein domains (0.60%). The biological functions of this network primarily centered around cellular response to molecules of bacterial origin, cellular response to biotic stimulus, response to lipopolysaccharide, regulation of inflammatory response, positive regulation of DNA-binding transcription factor activity, adaptive immune response based on somatic recombination of immune receptors built from immunoglobulin superfamily domains, and leukocyte chemotaxis (Figure 10 and Supplementary Material S1). S100A8 appears to occupy a central role within the interaction network, as evidenced by its involvement in numerous biological functions. These findings suggest that pathways related to inflammatory response and leukocyte chemotaxis may collaboratively contribute to the pathogenesis of acute pancreatitis, underscoring the need for further investigation into the specific pathway involved.

### Single-gene GSEA and GSVA of signature genes

Given the potential significance of S100A8, IL18, and AIF1 in the chemokine-related and Netosis-related pathway for AP and their role in regulating SAP, we chose to individually examine these three signature genes through single-gene GSEA and GSVA. Our findings were broadly in line with previous research.

**FIGURE 11:**
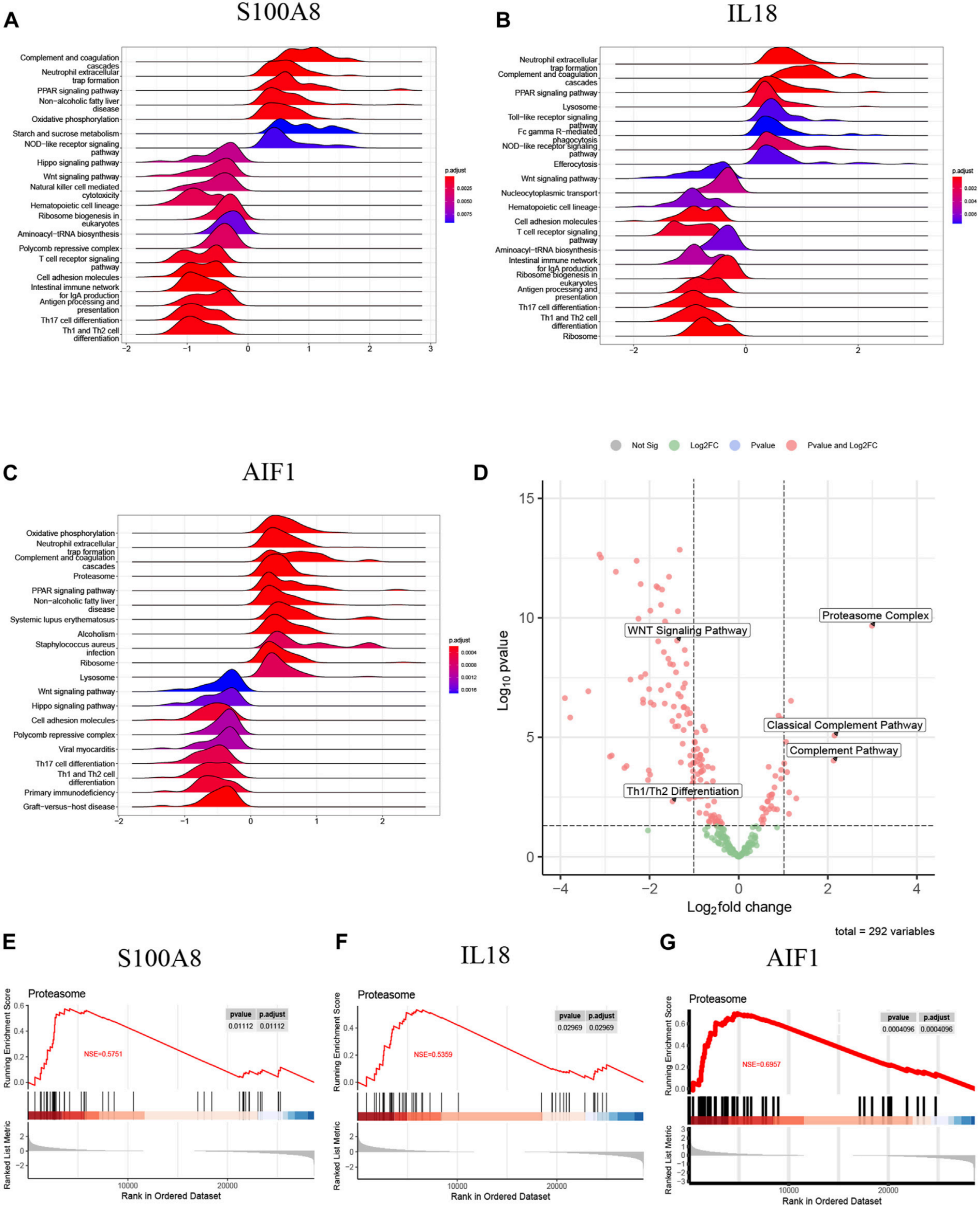
**(A)** The top 20 regulated KEGG pathway ranked by enrichmentscore from single-gene GSEA of S100A8; **(B)** The top 20 regulated KEGG pathway ranked by enrichmentscore from single-gene GSEA of IL18; **(C)** The top 20 regulated KEGG pathway ranked by enrichmentscore from single-gene GSEA of AIF1; **(D)** The GSVA-based volcano plot of upregulated and downregulated pathways between AP and healthy control groups in GSE194331; **(E–G)** The proteasome complex from single-gene GSEA of S100A8, IL18 and AIF1.

The samples of AP patients and healthy controls were categorized into high-expression and low-expression groups based on the mRNA levels of signature genes. Following this, a single-gene GSEA was performed using the KEGG pathways. Figure 11A illustrates that the S100A8 high-expression group exhibited upregulated activities in complement and coagulation cascades, neutrophil extracellular trap formation, and the PPAR signaling pathway. The downregulation of Th1/Th2 differentiation, T cell receptor signaling pathway, Wnt signaling pathway, and Th17 cell differentiation was observed in comparison.

Additionally, IL18 (Figure 11B) and AIF1 (Figure 11C) upregulation correlated with increased activity in neutrophil extracellular trap formation, complement and coagulation cascades, and PPAR signaling pathway. Furthermore, the decreased expression of IL18 (Figure 11B) and AIF1 (Figure 11C) is associated with reduced activity in the Th1 and Th2 differentiation, the Wnt signaling pathway, and Th17 cell differentiation. These pathways will be candidates for further validation.

**FIGURE 12:**
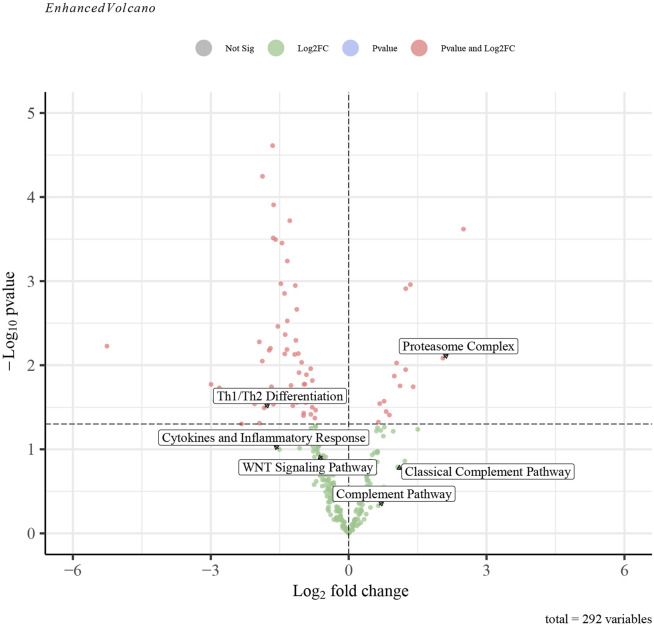
The GSVA-based volcano plot of upregulated and downregulated pathways between SAP and non-SAP groups in GSE194331.

The GSVA analysis investigated the distinctively regulated pathways between samples from individuals with AP and those from healthy controls. The findings of the GSVA analyses demonstrated that the Wnt signaling pathway and Th1 and Th2 differentiation activities were concurrently decreased in AP samples (Figure 11D). Similarly, the proteasome complex and complement pathway activities were concurrently increased in AP samples (Figure 11D). Consistently, in the context of GSEA, the activities of the proteasome complex were found to be concurrently increased due to perturbations in S100A8, IL18, and AIF1 (Figures 11E–G). Furthermore, the GSVA results for samples with SAP and non-SAP revealed that only the activities of the proteasome complex and Th1/Th2 differentiation exhibited a consistently regulated trend, as previously described (Figure 12).

## Discussion

As an inflammatory pancreatic disorder, AP is one of the more common acute abdominal diseases (Zheng et al., 2022), characterized by acute abdominal pain and increased concentrations of serum amylase and lipase. The pathological response of AP is cell death and inflammation (Zhan et al., 2019). Numerous researchers have endeavored to elucidate the pathogenesis of AP; moreover, the precise molecular mechanisms still need to be understood. Growing evidence suggests that SAP is intricately linked to tissue damage and disturbances in microcirculation, which arise from the release of proinflammatory cytokines and mediators such as TNF-α, IL-1β, IL-6, and intercellular adhesion molecule (ICAM)-1 (He et al., 2015). Experiments have shown that infiltrating inflammatory cells is crucial to experiment-induced AP by producing inflammatory mediators such as IL-1β and monocyte chemoattractant protein-1 (MCP-1) (Shen et al., 2018). Consequently, diminishing the concentrations of proinflammatory cytokines to impede the inflammatory cascade may present a viable therapeutic approach for AP (*29*).

As a hallmark of AP, neutrophils play a pivotal role in SAP and contribute to acute lung injury (Guo and Cui, 2018; Murthy et al., 2019; Andersson et al., 2020). A significant correlation exists between neutrophil migration and chemokine gradients (Kim et al., 2017), with the concentration of chemotactic factors guiding neutrophils toward the injury site (Daseke et al., 2021). Prior studies have demonstrated that depleting or inhibiting neutrophils can protect against pancreatic tissue damage in inflammatory responses reliant on neutrophil activity (Cui et al., 2017). Neutrophil-induced NETosis is a distinct form of neutrophil death that differs from apoptosis and necrosis and significantly contributes to tissue damage (Liu et al., 2022; Wang C. L. et al., 2023). By responding to chemokines, activated neutrophils migrate into the site of inflammation, produce antimicrobial agents, and undergo NETosis (Balachandran et al., 2022). Thus, chemokines are crucial regulators in the process of NETosis. However, the involvement of NETosis, conducted by chemokines, in the mechanistic pathways of AP remains uncertain.

As previously mentioned, chemokine-regulated NETosis may play a crucial role in the pathogenesis of AP, particularly SAP, characterized by dysregulated inflammatory response and tissue damage. In this study, we first analyzed to identify DEGs between AP and healthy control samples. A total of 565 DEGs were identified, and GO enrichment analyses revealed significant involvement of processes such as migration, chemotaxis, activation of neutrophil, and chemokine binding. These results align with our initial hypotheses and warrant further investigation.

Moreover, we have identified 12 DECRGs and 7 DENRGs that are primarily enriched in functions related to chemokine, cytokine, inflammatory, and immune pathways, such as the NOD-like receptor signaling pathway and TNF signaling pathway. TNF-α is a proinflammatory cytokine that triggers inflammation and pain in conditions like AP, hepatitis, and inflammatory bowel disease. Inhibiting TNF-α has been shown to improve outcomes in experimental AP (Oz, 2016). Thus, it is reasonable to infer that the varied expression of NETosis and chemokine-related genes may play a role in the pathogenesis of AP.

Machine learning algorithms demonstrate superior performance compared to standard logistic regression in developing accurate classification and prediction models for diseases (Alonso-Betanzos and Bolón-Canedo, 2018). An integrated algorithm incorporating LASSO, SVM-RFE, and RF was employed to identify signature genes with the highest predictive accuracy. Subsequently, three signature genes (S100A8, IL18, and AIF1) were identified as the final selection from the intersection of featured genes. Two nomogram models were developed and validated for their predictive efficacy using calibration curves, DCA, and CIC. The ROC curve was utilized to assess the performance of these nomogram models, yielding an AUC of 0.968 (95% CI, 0.937–0.990) for predicting AP and 0.862 (95% CI, 0.742–0.955) for predicting SAP. In conclusion, the findings indicated that S100A8, IL18, and AIF1 play a critical role in NETosis and chemokine regulation in AP, including SAP, and the diagnostic models utilizing these genes demonstrated high efficacy.

Within the S100 protein family, S100A8 and S100A9 predominantly form heterodimers (S100A8/A9; calprotectin) *in vivo* (Kotani et al., 2022). Previous studies demonstrated that S100A8 has been utilized as a surrogate marker for neutrophil activity, assessed by myeloperoxidase (MPO) levels (Espira et al., 2023). There is evidence that S100A8 is a neutrophil-specific chemokine (Wu et al., 2022). Through immunocytochemistry, Sprenkeler EGG et al. found S100A8/A9 in NETs and demonstrated that it was released simultaneously with DNA during NETosis (Sprenkeler et al., 2022). Activated neutrophils immediately release S100A8/A9 upon activation, making it an ideal biomarker for rapid inflammatory responses (Jonasson et al., 2017). S100A8/A9 was significantly increased in exosomes from SAP and activated NADPH oxidase, producing free radicals and promoting inflammatory response (Carrascal et al., 2022). Exhilaratingly, our findings demonstrated that the increased expression of S100A8 is significantly correlated with the severity of AP and the KEGG pathway of “Neutrophil extracellular trap formation”, aligning with existing literature and bolstering our hypothesis.

IL18 was first described as an endotoxin-induced serum factor. As part of the IL1 family, IL18 can stimulate systemic and local inflammation by releasing TNF-α, COX2, and GM-CSF (Wang X. et al., 2023). Emerging research indicated that IL-18 plays a significant regulatory role in immunoregulation across various inflammatory and malignant conditions. Several studies have shown a link between IL18 and cancer, specifically in pancreatic ductal adenocarcinoma (PDAC), where high levels of IL18 are associated with increased mortality and poor disease outcomes (Yamanishi et al., 2023). Recent studies have elucidated the involvement of IL-18 in pancreatic disorders, presenting novel opportunities for therapeutic strategies that target this cytokine (Li et al., 2019). A notable elevation in serum IL-18 levels was observed within 24 h of symptom onset in patients with AP compared to healthy individuals. Furthermore, the concentration of this cytokine was significantly higher in patients with SAP compared to those with mild cases (Rotstein, 2014; Sternby et al., 2016; Li et al., 2019; Ćeranić et al., 2020). Additionally, the absence of IL-18 significantly decreased neutrophil infiltration in the injured pancreas and impaired neutrophil maturation in the spleen of mice with SAP (Li et al., 2019).

Therefore, the high expression of IL18 may be associated with poor prognosis in AP and PDAC. Our investigation observed a notable elevation in serum IL18 expression in the AP group, particularly in the SAP subgroup. This increase in IL18 levels was associated with heightened activity in the KEGG pathway term “Neutrophil extracellular trap formation” via single-gene GSEA. These findings align with prior studies and might demonstrate the remarkable regulatory function of IL18 in AP.

Allograft Inflammatory Factor-1 (AIF1) is expressed in myeloid cells and functions to enhance immune responses and regulation (da Silva et al., 2021). There is evidence that AIF1 can regulate monocyte/macrophage, microglia, and lymphocyte immune activity, and it may stimulate these cells to produce cytokines, chemokines, and nitric oxide synthase (Liu et al., 2018). Also, AIF1 plays a critical role in migration, phagocytosis, proliferation, survival, and pro-inflammatory activity in macrophages (da Silva et al., 2021). Our study revealed a significant increase in AIF1 levels in the AP group, particularly in the SAP subgroup, and a significant positive correlation with activating the kEGG pathway “Neutrophil extracellular trap formation”. However, a paucity of literature supports the association and underlying mechanism of AIF1 with NETosis and AP. Additional clinical and mechanistic investigations are warranted to validate these findings.

The results of single-gene GSEA demonstrated a noteworthy association between the upregulated genes S100A8, IL18, and AIF1 and heightened activity of the “Proteasome Complex.” This association was consistently observed in both the AP and SAP groups, as indicated by GSVA. The proteasome plays a crucial role in NF-κB activation and can potentially modulate the development of inflammatory conditions (Zhu et al., 2018). Dong X et al. illustrated in their study that the proteasome inhibitor PS-341 effectively mitigated the severity of acute pancreatitis induced by cerulein and lipopolysaccharide in mice. This observed effect is attributed to the inhibition of NF-κB activation, leading to improvements in various parameters, including serum amylase, CRP, lactate dehydrogenase, IL-1β, IL-6, and pancreatic MPO levels (Dong et al., 2010). Similarly, Zhu QT documented that the proteasome plays a role in the development of AP and that its inhibitor, bortezomib, demonstrated protective effects against AP in mice by reducing the expression of NF-κB p65 nucleoprotein and total proteasome 20S protein (Zhu et al., 2018).

Conversely, the perturbation of S100A8, IL18, and AIF1 resulted in the downregulation of the activity of “Th1 and Th2 cell differentiation”. This downregulation was observed not only in the AP group but also in the SAP subgroup. It has been proposed that a Th1 profile is linked to SAP, while a Th2 profile is associated with MAP or MSAP (Rodriguez-Nicolas et al., 2018). However, another study found that the analysis of spleen-derived cells from mice with acute pancreatitis showed increased levels of Th2 cells, while Th1 cells remained unchanged (Sendler et al., 2020). Furthermore, research indicated that the quantity and function of circulating Th1 and Th2 cells undergo dynamic alterations, with the Th1/Th2 ratio playing a significant role in determining the progression and severity of AP (Stojanovic et al., 2023). Hence, the diverse and dynamic changes in “Th1 and Th2 cell differentiation” in AP necessitate further comprehensive investigation.

Indeed, this study was subject to limitations, notably just retrospective and data-based analyses. Secondly, inflammation-related molecules are susceptible to modulation by a multitude of environmental and genetic factors. The expression levels of inflammatory cytokines and chemokines may exhibit significant variability in response to differing physiological states within the same patient population. The upregulation of these three genes in AP, particularly in SAP, is just a subject of speculation at the transcriptome level. The precise relationship between this upregulation and AP and its role in specifically determining the severity of AP and the specific molecular mechanisms remains unclear. Thus, further large-sample clinical surveys and experimentation based on appropriate cell/animal models and proteomics are necessary to validate our findings.

However, our study, for the first time, screened and verified that chemokines and NETosis-related feature genes could be used to construct effective prediction models for AP and SAP. These outcomes might provide an emerging perspective on the association between NETosis and AP and contribute to revealing the novel therapeutic target of AP. Furthermore, it is noteworthy that the diagnostic models utilizing three signature genes were also effective in the internal test group, showing a good performance in distinguishing SAP from non-SAP, thereby bolstering the reliability of our results. The integration of these signature genes with existing clinical diagnostic models could be a worthwhile endeavor.

## Conclusion

Through the utilization of integrated bioinformatics and various machine-learning algorithms, we have identified three distinct chemokine-related and NETosis-related genes (S100A8, IL18, and AIF1). Additionally, we have developed accurate diagnostic models for identifying AP, focusing on SAP. This novel approach might contribute significantly to AP’s clinical diagnosis and treatment.

## Data Availability

Publicly available datasets were analyzed in this study. This data can be found here: https://www.ncbi.nlm.nih.gov/geo/query/acc.cgi?acc=GSE194331. Further inquiries can be directed to the corresponding authors.
