# Supplementary material for: Identification and analysis of chemokine-related and NETosis-related genes in acute pancreatitis to develop a predictive model
Source: Front Genet. 2024 May 9;15:1389936. doi: 10.3389/fgene.2024.1389936 (PMC11112067; doi:10.3389/fgene.2024.1389936)
Supplement: Supplementary file 1 [file DataSheet1.ZIP › supplementary materials/Supplementary figure 1_ KEGG_pathways enrichment analysis of DECRGs and DENRGs.pdf]

Supplementary figure1

A

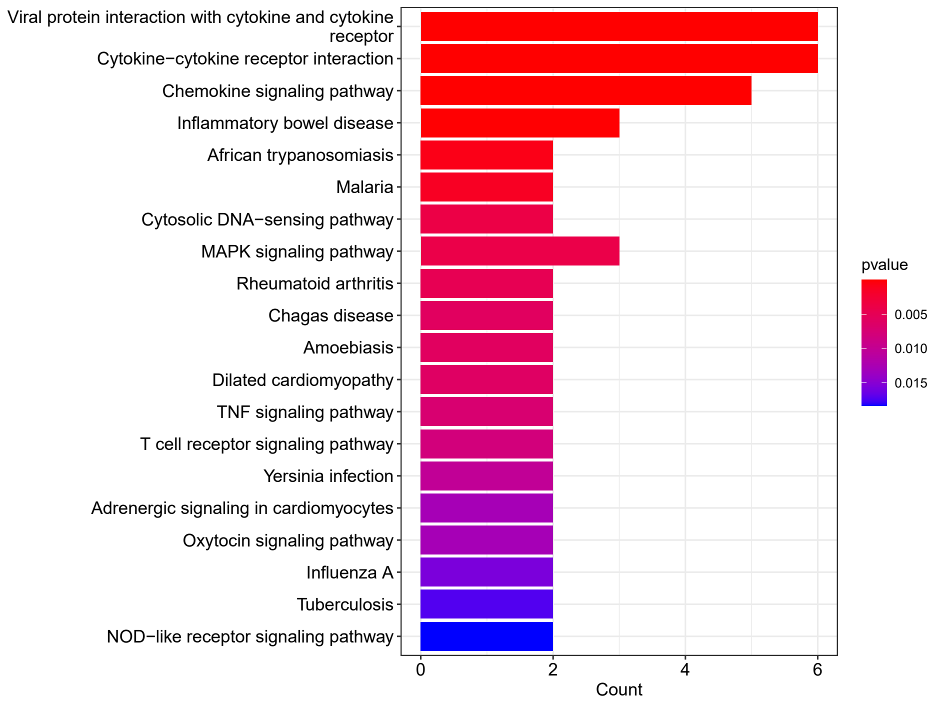

KEGG pathways enrichment analysis of DECRGs

B

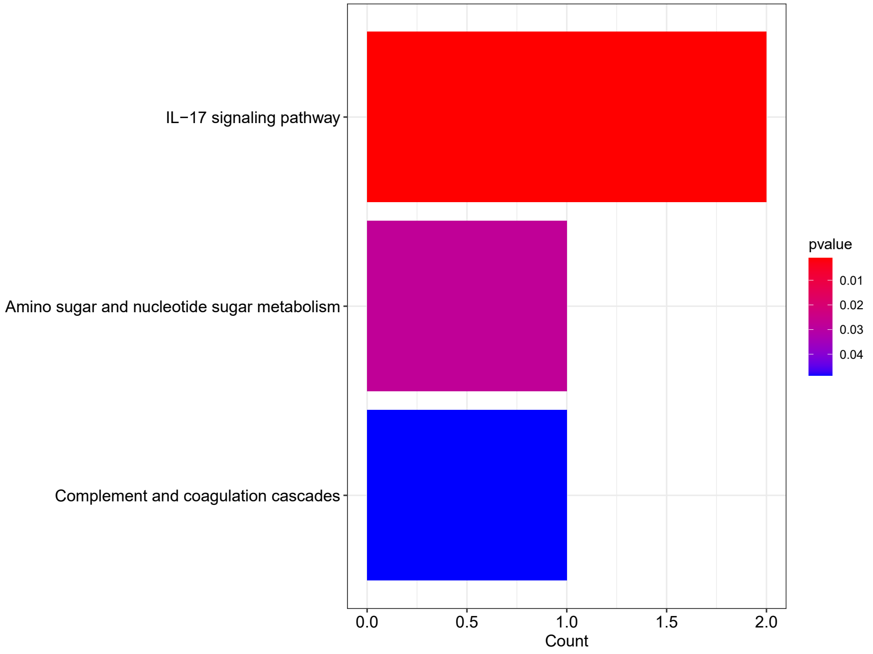

KEGG pathways enrichment analysis of DENRGs

Supplementary figure 1_ KEGG_pathways enrichment analysis of DECRGs and DENRGs
